# Cardiac adipose tissue volume and IL-6 level at admission are complementary predictors of severity and short-term mortality in COVID-19 diabetic patients

**DOI:** 10.1186/s12933-021-01327-1

**Published:** 2021-08-12

**Authors:** Franck Phan, Samia Boussouar, Olivier Lucidarme, Mohamed Zarai, Joe-Elie Salem, Nadjia Kachenoura, Khaoula Bouazizi, Etienne Charpentier, Yasmine Niati, Hasnae Bekkaoui, Zahir Amoura, Alexis Mathian, Olivier Benveniste, Patrice Cacoub, Yves Allenbach, David Saadoun, Jean-Marc Lacorte, Salma Fourati, Suzanne Laroche, Agnes Hartemann, Olivier Bourron, Fabrizio Andreelli, Alban Redheuil, COVID-19 APHP.SU Group

**Affiliations:** 1 Sorbonne Université, Paris, France; 2 Assistance Publique-Hôpitaux de Paris (APHP), Diabetology Department, La Pitié Salpêtrière-Charles Foix University Hospital, Paris, France; 3 Centre de Recherche des Cordeliers, INSERM, UMR_S 1138, Paris 06, France; 4 Institute of Cardiometabolism and Nutrition ICAN, Paris, France; 5 Cardiovascular and Thoracic Imaging Unit, Hôpital Pitié Salpêtrière, Assistance Publique-Hôpitaux de Paris (APHP), Sorbonne Université, Paris, France; 6 Laboratoire d’Imagerie Biomédicale, Sorbonne Université, INSERM, CNRS, Institute of Cardiometabolism and Nutrition, Paris, France; 7 Service d’imagerie Spécialisée et d’urgence SISU, Hôpital Pitié Salpêtrière, Assistance Publique-Hôpitaux de Paris, Laboratoire d’Imagerie Biomédicale, Sorbonne Université, INSERM, CNRS, Paris, France; 8 Department of Pharmacology, CIC-1901, INSERM, Sorbonne Université, Assistance Publique-Hôpitaux de Paris (APHP), Paris, France; 9 Service de Médecine Interne 2, Centre National de Référence Maladies Systémiques Rares et Histiocytoses, Institut e3M, Hôpital de La Pitié-Salpêtrière, AP-HP, Sorbonne Université, 75013 Paris, France; 10 Département de Médecine Interne et Immunologie Clinique, Hôpital Pitié-Salpêtrière, Sorbonne Université, AP-HP, Paris, France; 11 Department of Endocrine and Oncologic Biochemistry, Inserm, UMR_S 1166, Research Institute of Cardiovascular Disease, Metabolism and Nutrition, Paris, France; 12 Nutrition and Obesities: Systemic Approaches (NutriOmics) Research Unit, Sorbonne Université, INSERM, UMRS U1269, Paris, France

**Keywords:** Cardiac computed tomography, Cardiac adipose tissue, Inflammation, Interleukin-6, COVID-19, Diabetes, Outcome

## Abstract

**Background:**

COVID-19 diabetic adults are at increased risk of severe forms irrespective of obesity. In patients with type-II diabetes, fat distribution is characterized by visceral and ectopic adipose tissues expansion, resulting in systemic inflammation, which may play a role in driving the COVID-19 cytokine storm. Our aim was to determine if cardiac adipose tissue, combined to interleukin-6 levels, could predict adverse short-term outcomes, death and ICU requirement, in COVID-19 diabetic patients during the 21 days after admission.

**Methods:**

Eighty one consecutive patients with type-II diabetes admitted for COVID-19 were included. Interleukin-6 measurement and chest computed tomography with total cardiac adipose tissue index (CATi) measurement were performed at admission. The primary outcome was death during the 21 days following admission while intensive care requirement with or without early death (ICU-R) defined the secondary endpoint. Associations of CATi and IL-6 and threshold values to predict the primary and secondary endpoints were determined.

**Results:**

Of the enrolled patients (median age 66 years [IQR: 59–74]), 73% male, median body mass index (BMI) 27 kg/m^2^ [IQR: 24–31]) 20 patients had died from COVID-19, 20 required intensive care and 41 were in conventional care at day 21 after admission. Increased CATi and IL-6 levels were both significantly related to increased early mortality (respectively OR = 6.15, p = 0.002; OR = 18.2, *p* < 0.0001) and ICU-R (respectively OR = 3.27, *p* = 0.01; OR = 4.86, *p* = 0.002). These associations remained significant independently of age, sex, BMI as well as troponin-T level and pulmonary lesion extension in CT. We combined CATi and IL-6 levels as a multiplicative interaction score (CATi*IL-6). The cut-point for this score was ≥ 6386 with a sensitivity of 0.90 and a specificity of 0.87 (AUC = 0.88) and an OR of 59.6 for early mortality (*p* < 0.0001).

**Conclusions:**

Cardiac adipose tissue index and IL-6 determination at admission could help physicians to better identify diabetic patients with a potentially severe and lethal short term course irrespective of obesity. Diabetic patients with high CATi at admission, *a fortiori* associated with high IL-6 levels could be a relevant target population to promptly initiate anti-inflammatory therapies.

**Supplementary Information:**

The online version contains supplementary material available at 10.1186/s12933-021-01327-1.

## Background

COVID-19 diabetic adults are at increased risk for hospital admission, development of severe forms requiring intensive care (ICU-R) and death irrespective of obesity [1]. In patients with type 2 diabetes, fat distribution is characterized by visceral (VAT) and ectopic adipose tissues expansion, resulting in an increased interleukin 6 (IL-6) release and systemic low-grade inflammation. To what extent VAT expansion and the associated ectopic fat depots, may play a role in driving the COVID-19 cytokine storm is still discussed [2–4]. Among ectopic adipose tissues, increased epicardial adipose tissue was shown to predispose individuals with coronary artery disease to accelerated atherosclerosis, sudden death and other cardiovascular events in diabetic patients. Considering the reported cardiac involvement including acute coronary syndrome, myocarditis and heart failure among COVID-19 patients [5], we aimed at analyzing if cardiac adipose tissue, combined to plasmatic inflammatory markers, could predict the short-term outcomes (ICU-R or death during the 21 days after admission) in COVID-19 diabetic patients.

## Methods

Eighty one consecutive patients with type-II diabetes admitted for COVID-19 (positive SARS-CoV-2 PCR) in a large tertiary care academic center between March 1st and April 30th 2020 were included in the study. Blood samples at admission included glycemia, lymphocyte and platelet count, C-Reactive Protein, Interleukin-6 (IL-6), procalcitonin, D-Dimers, troponin-T, fibrinogen and glomerular filtration rate estimation. Chest computed tomography (CT) was performed at admission to evaluate COVID-19 pneumonia severity using a scale of pulmonary parenchyma lesions extension.

During follow-up, patients remained in a conventional medical unit (CMU) or were admitted to an ICU as required according to clinical severity criteria. This monocentric observational study was based on a COVID-19 cohort approved by the local ethics committee (CER-SU 2020-14) and registered as NCT04320017. According to local legislation all study participants could withdraw their participation in the study.

**Fig. 1:**
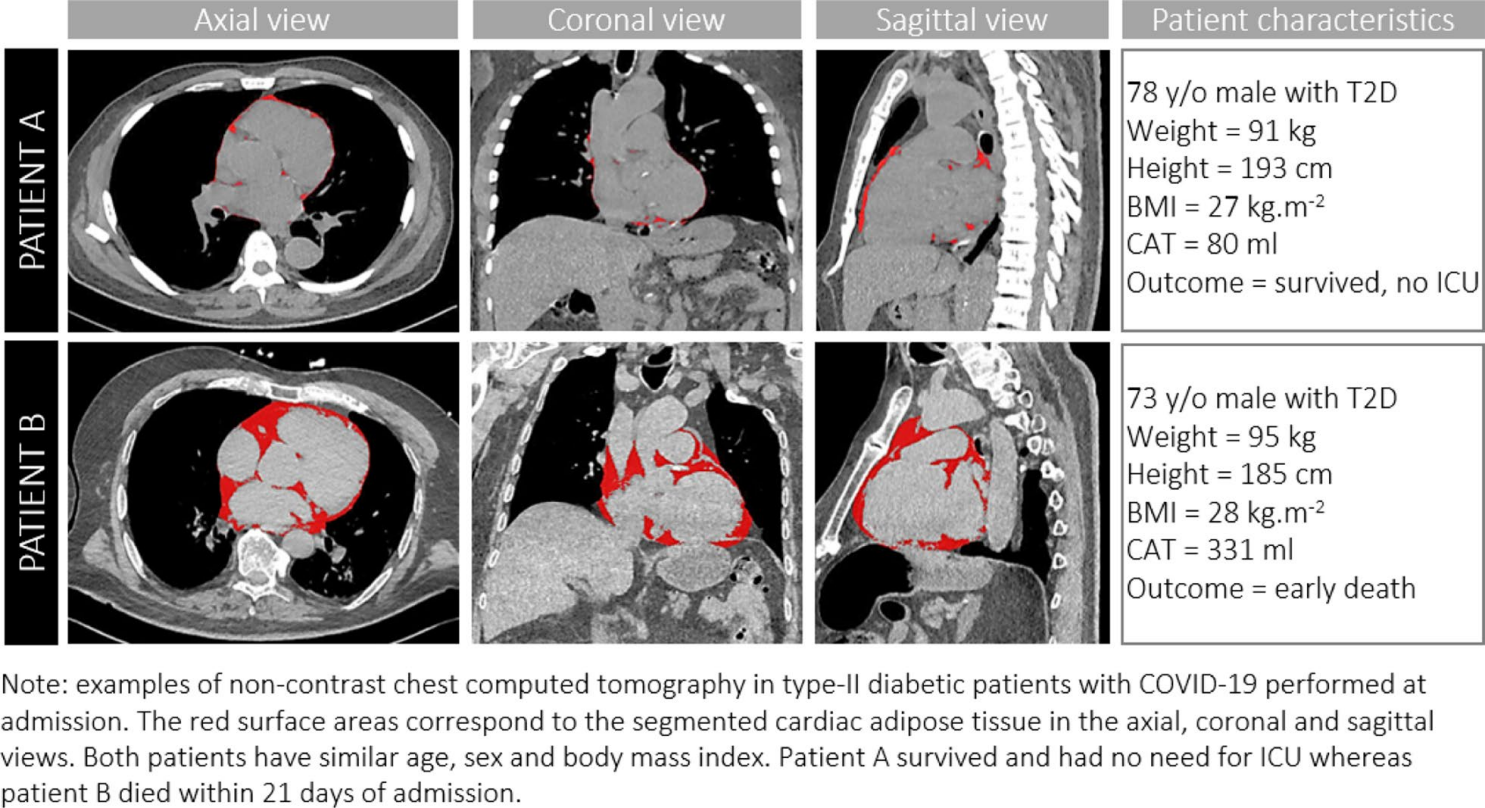
Quantification of cardiac adipose tissue on chest computed tomography and illustration of study results

Cardiac adipose tissue (CAT) volumes were determined from CT and indexed to body surface area (CATi). Non ECG-gated non contrast helicoidal thoracic acquisitions were performed at 120 kV with 0.6 mm collimation on either a dual source SOMATOM Definition Flash or EDGE scanner (Siemens Healthineers). Total cardiac adipose tissue included epicardial and pericardial fat and was measured using an automated segmentation method (Siemens Frontier) with thresholding centered on the density range of adipose tissue values (-150 to -30 Hounsfield Units). Segmentation results of typical CAT patterns are illustrated on Fig. 1 in relation to outcomes of two individuals otherwise comparable for age, gender, height, weight and BMI. Lung involvement was measured semi-quantitatively and reported according to the parenchymal extension of lung lesions (ground glass and/or condensation) as: minimal (< 10%), moderate (25–50%), extensive (25–50%) and severe (> 50%).

### Statistical analysis

The primary outcome was death during the 21 days following admission while intensive care requirement with or without early death (ICU-R), summarizing clinical disease severity, defined the secondary endpoint. Median and frequency differences between groups were assessed using Mann–Whitney rank-sum and chi2 tests, respectively. Associations of CAT index and inflammatory markers with outcomes were studied using univariate and multivariate logistic regression models adjusting for age, sex and BMI. Further adjustment for troponin level was also reported as it was the biomarker with the second strongest relationship to outcomes after IL-6. Maximization of the sensitivity and specificity product was used to determine threshold values for CAT index and IL-6 levels to predict the primary and secondary endpoints. An integrated score was calculated as the interaction term between CAT index and IL-6 defined as CATi*IL-6 and its relationship to outcomes was studied using logistic regression as mentioned above. Receiver operating characteristic analysis was performed to compare the classifying performance of CATi, IL-6 and CATi*IL-6 with regard to outcomes and corresponding ROC-curves and area-under-curve values (AUC) are provided. *P* < 0.05 was considered statistically significant.

## Results

Baseline characteristics of the enrolled patients (median age 66 years [IQR: 59–74]), 73% male, median body mass index (BMI) 27 kg/m^2^ [IQR: 24–31]) are summarized in Table 1. At day 21 after admission, 20 patients had died from COVID-19, 20 required intensive care and 41 were in CMU. Early mortality was associated with a higher proportion of patients having hypertension (*p* = 0.02) and dyslipidemia (*p* = 0.02) whereas there was no difference between groups in smoking status. There was a higher proportion of patients with dyslipidemia in the severe outcome group (early death and ICU requirement) compared to the conventional medical care group (Table 1).

**Table 1:** Baseline characteristics, imaging and biological data according to outcomes at Day 21 after admission for COVID-19

	Survivors	Death	(p)	CMU	ICU + Death	(p)
	n = 61 (75%)	n = 20 (25%)		n = 41 (50.6%)	n = 40 (49.4%)	
Anthropometry						
Age, years (IQR)	65 (56–72)	71 (67–78)	0.02	66 (57–77)	69 (59–73)	0.99
Male, n(%)	44 (72)	15 (75)	0.8	27 (66)	31 (77.5)	0.32
Weight (kg)	79 (66–90)	79 (69–89)	0.73	76 (65–90)	79 (69–89)	0.46
BMI (kg/m^2^)	26 (24–31)	28 (25–31)	0.32	25.7 (22–31)	27.3 (25–31)	0.19
Cardiometabolic risk profile						
Glycemia, mmol/l	8.4 (6.9–10.8)	10.6 (8.9–13.2)	0.03	8.2 (6.8–10.3)	9.9 (7.3–13.1)	0.03
Active smoking, n(%)	6 (9.8)	1 (6.7)	0.69	4 (9.7)	3 (7.5)	0.83
Hypertension, n(%)	42 (68.8)	19 (95)	0.02	30 (73.1)	31 (77.5)	0.65
Dyslipidemia, n(%)	24 (55.7)	17 (85)	0.02	21 (51.2)	30 (75)	0.03
Insulin therapy, n(%)	24 (39.3)	9 (45)	0.65	17 (41.5)	16 (40)	0.89
Quantitative Imaging biomarkers						
Cardiac adipose tissue (mL)	216 (138–280)	259 (215–337)	0.007	183 (126–265)	248 (200–340)	0.005
Cardiac adipose tissue (mL/m^2^)	112 (72–150)	148 (121–178)	0.006	106 (72–144)	138 (104–171)	0.01
Lung CT scan severity score			0.22			0.08
Minimal (10%)	8 (12)	5 (25)		7 (17)	6 (15)	
Moderate (10–25%)	18 (29)	4 (20)		15 (37)	7 (17)	
Extensive (25–50%)	24 (40)	10 (50)		16 (39)	17 (43)	
Severe (> 50%)	11 (19)	1 (5)		3 (7)	10 (25)	
Inflammatory markers						
Interleukin 6 (pg/mL)	25 (10–41)	56.3 (50.8–98.2)	< 0.001	24.3 (8.5–42)	49 (25–56)	0.002
C-reactive protein (mg/L)	87 (38–137)	67 (36–157)	0.87	84.5 (37–120)	89 (41–168)	0.46
Procalcitonin (μg/L)	0.16 (0.09–0.26)	0.3 (0.1–0.5)	0.04	0.15 (0.1–0.32)	0.2 (0.1–0.35)	0.09
Fibrinogen (g/L)	6.5 (5.7–7.5)	6.6 (5.3–7.7)	0.94	6.4 (5–7)	6.7 (5.6–7.7)	0.29
Leukocytes (× 10^9^/L)	6.4 (5–7.9)	8.3 (6.1–8.9)	0.02	6.3 (5–8)	7.7 (5.6–9)	0.06
Lymphocytes (× 10^9^/L)	1.1 (0.8–1.4)	0.94 (0.7–1.3)	0.11	1.1 (0.9–1.5)	0.94 (0.7–1.3)	0.04
Other biomarkers						
D-dimers (ng/mL)	825 (545–1775)	1405 (680–2430)	0.10	715 (477–1513)	1340 (718–2310)	0.009
T-Troponin (ng/L)	13.4 (9.4–34.2)	45.2 (29.1–89.5)	0.001	14 (9–32)	33 (12–51)	0.007
Platelets (× 10^9^/L)	220 (166–291)	201 (161–219)	0.06	220 (164–293)	205 (177–269)	0.4
AST (UI/L)	59 (29–67)	52 (37–79)	0.53	43 (28–67)	49 (34–80)	0.48
ALT (UI/L)	35 (22–62)	31 (20–38)	0.19	31 (20–59)	35 (20–55)	0.82
eGFR (mL/min/1.73m^2^)	72 (49–98)	69 (47–77)	0.22	72 (49–101)	71.5 (47–88)	0.60

*CMU* conventional medical care unit, *ICU* intensive care unit. Data are represented as median (interquartile range) and frequency (percent) as appropriate. Median and frequency differences were tested using Mann–Whitney and chi^2^ tests, respectively

Age, leukocytes, procalcitonin and troponin-T levels at admission were significantly higher in patients with early mortality compared to the CMU group (Table 1). Troponin and D-dimers were higher and leukocyte count reduced in the ICU-R group compared to the CMU group (Table 1). Glycemia was increased in patients with early mortality and ICU-R compared to the CMU group (both *p* = 0.03). Lung severity score in CT did not differ significantly across groups according to outcome.

### Relationship of cardiac adipose tissue and IL-6 to outcomes

**Fig. 2:**
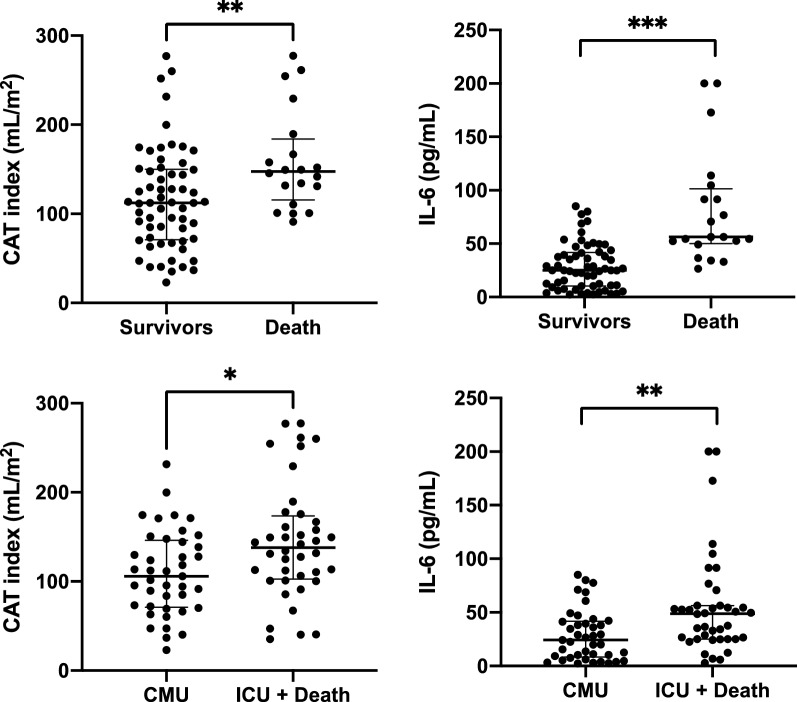
Cardiac adipose tissue volume index and IL-6 levels distributions according to short-term outcomes after 21 days of follow up. Note: *CMU* conventional medical care unit, *ICU* intensive care unit, *CAT* Cardiac adipose tissue, *IL-6* Interleukin-6. Data are represented as median with interquartile range. Median difference was tested using Mann–Whitney test. p: **: < 0,01; *** < 0,001

Increased CATi was related to early mortality both as a continuous variable (*p* = 0.006) and as a dichotomous variable (OR = 6.15, *p* = 0.002; Fig. 2, Tables 1,2). IL-6 levels were significantly higher in patients with early mortality both as continuous (*p* < 0.0001) and dichotomous variables (OR = 18.2, *p* < 0.0001; Fig. 2, Table 2). In multivariate analysis, both increased CATi (OR = 5.2, *p* = 0.01) and IL-6 (OR = 17, *p* < 0.0001) were significantly associated with early mortality independently of age, sex and BMI. These relationships were also significant when considering CATi and IL-6 as continuous variables and remained significant even after further adjustment for troponin-T or extensive/severe lung involvement in CT.

**Table 2:** Univariate and Multivariate Relationship of Cardiac Adipose Tissue Index and IL6 with outcomes at Day 21 in diabetic patients after admission for COVID-19

	Univariate analysis			Multivariate analysis*		
	OR	95% CI	(p)	OR	95% CI	(p)
**Early mortality**						
*Test of individual variables*						
CATi (continuous variable)	1.01	1.003–1.02	0.006	1.01	1.001–1.02	0.033
CATi (increased ≥ 130 ml/m^2^)	6.15	1.96–19.3	0.002	5.20	1.46–18.38	0.011
IL6 (continuous variable)	1.06	1.03–1.09	< 0.0001	1.05	1.02–1.09	0.001
IL6 (increased ≥ 49 pg/ml)	18.2	5.07–65.09	< 0.0001	17.0	4.40–65.9	< 0.0001
*Test of CATi + IL6 as covariates*						
CATi (continuous variable)				1.02	1.01–1.04	0.003
IL6 (continuous variable)				1.07	1.03–1.12	< 0.0001
Test of integrated score CATi*IL6						
CATi*IL6 score (increased ≥ 6386)	59.6	11.6–307.1	< 0.0001	58.1	9.8–344.7	< 0.0001
**ICU + Death**						
*Test of individual variables*						
CATi (continuous variable)	1.01	1.003–1.02	0.01	1.01	1.001–1.02	0.038
CATi (increased ≥ 130 ml/m^2^)	3.27	1.30–8.20	0.01	3.12	1.12–8.68	0.029
IL6 (continuous variable)	1.03	1.006–1.04	0.01	1.02	1.003–1.04	0.024
IL6 (increased ≥ 49 pg/ml)	4.86	1.75–13.51	0.002	4.59	1.58–13.38	0.005
*Test of CATi + IL6 as covariates*						
CATi (continuous variable)				1.01	1.002–1.02	0.016
IL6 (continuous variable)				1.03	1.006–1.05	0.011
Test of integrated score CATi*IL6						
CATi*IL6 score (increased ≥ 6386)	7.96 (3.62)	2.59–24.45	< 0.0001	9.02 (3.49)	2.62–31.06	< 0.0001

^*^Multivariate logistic regression with adjustment for age, sex and BMI

CATi (OR = 3.27, p = 0.01) and IL-6 (OR = 4.86, *p =* 0.002) were both significantly increased in ICU-R patients (Fig. 2, Tables 1,2). These relationships were also significant when considering CATi and IL-6 as continuous variables. These associations remained significant for CATi (OR = 3.12, *p* = 0.03) and IL-6 (OR = 4.59, *p* = 0.005) independently of age, sex, BMI and also after further adjustment for troponin-T or extensive/severe lung involvement in CT.

When entered both as covariates in the adjusted model both CATi and IL-6 remained independently and significantly associated with early mortality and ICU-R (Table 2).

**Fig. 3:**
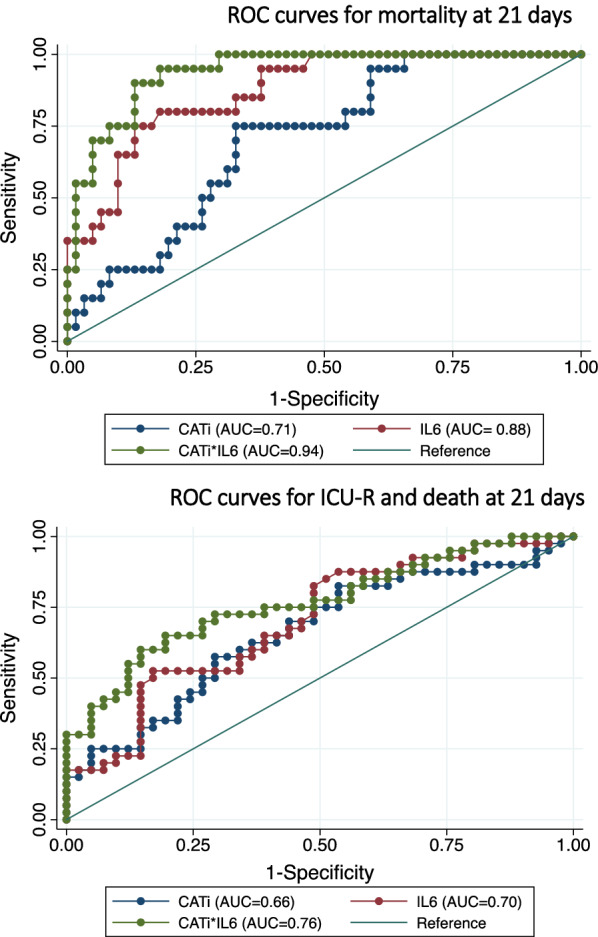
ROC analysis comparing the individual and combined performance of cardiac adipose tissue index and IL6 to predict adverse events in diabetic patients with COVID-19. *AUC* area under curve of the ROC analysis in parentheses in the figure legend. *CATi* cardiac adipose tissue index

Threshold values were determined for early outcome for both CAT index and IL-6. For early mortality, individual cut-points were CAT index ≥ 130 mL/m^2^ with sensitivity/specificity of 0.75/0.67, AUC = 0.71 and IL-6 ≥ 49 pg/mL with sensitivity/specificity of 0.80/0.82, AUC = 0.81 as shown on Fig. 3.

To improve the prediction of adverse outcomes, we combined CAT volume and IL-6 levels as a simple interaction score defined as: CATi*IL-6 (AUC = 0.94, Fig. 3). The optimal cut-point for this score was ≥ 6386 with a sensitivity of 0.90 and a specificity of 0.87 (AUC = 0.88) for early mortality (Fig. 3). An increased CATi*IL-6 score ≥ 6386 was strongly related to early mortality with OR = 58.1 (*p* < 0.0001) and ICU-R with OR = 9.02 (*p* < 0.0001) after adjustment for age, sex and BMI and also after further adjustment for troponin-T or extensive/severe lung involvement in CT (Table 2).

## Discussion

In this COVID-19 cohort of diabetic patients, elevated IL-6 levels and cardiac adipose tissue volume were independently associated with early mortality and a composite endpoint including ICU-R. Furthermore, we describe a simple integrative score, based on both parameters, demonstrating an optimized prediction value for both endpoints.

Adult diabetic patients with COVID-19 are at increased risk for hospital admission, development of severe forms requiring intensive care and death. Obesity, a widely known risk factor for cardiometabolic events, has also been associated with increased severity of COVID-19. Indeed, BMI is significantly correlated with mortality of COVID-19 suggesting that excess of fat mass may be the main driver of the SARS-CoV-2-induced cytokine storm [6, 7]. Nevertheless, BMI cannot distinguish visceral from subcutaneous fat while this distinction is key in the pathophysiology of metabolic syndrome, type 2 diabetes, NASH, risk for cardiovascular events and potentially COVID-19 susceptibility or severity [8]. Recently, Pranata et al*.* showed in a meta-analysis that visceral adiposity was associated with increased COVID-19 severity, while subcutaneous adiposity was not [9].

This important association can be explained by a variety of mechanisms, among which low grade inflammation, insulin resistance and hyperglycemia have been extensively studied.

Under long-term over-nutrition, VAT expansion promotes low-grade inflammation with local production of IL-6, TNFα, and IL-1. This is associated with a reduction of anti-inflammatory immune cells (Tregs and eosinophils) and the recruitment of pro-inflammatory cells: activated T-cells, IFNγ-producing natural killer (NK) cells, and inflammatory macrophages [10]. This local inflammatory milieu is an important etiological factor of the development of insulin resistance, dysfunction of insulin secretion and hyperglycemia [11]. The observation that hyperglycemia is more commonly observed in non survivors after ICU admission emphasizes the importance of abdominal fat as a relevant determinant of poor COVID-19 prognosis [12–14]. In agreement with the literature, we confirmed in our cohort that blood glucose levels at admission were significantly higher in non survivors and in patients reaching the composite endpoint including ICU-R. Surprisingly, most clinical cohorts failed to show that HbA1c levels at admission were related to COVID-19 outcome (see Additioanl file 1: Table S1). This suggests that acute stress-induced hyperglycemia and aggravation of pre-existing diabetes (rather than chronic high glucose levels) are determinants of short-term prognosis during COVID-19 disease. Among other factors, high glucose levels may activate pro-inflammatory immune cells and pro-inflammatory polarization of macrophages (M1) leading to exacerbation of the cytokine storm and consequently, poor outcome.

In diabetic patients, the VAT component is predominantly increased vs. subcutaneous fat contrary to what has been found in non-diabetic obese individuals using MRI-based abdominal adipose tissue quantification [15]. The expansion of VAT is usually associated with the development of other ectopic fat depots such as CAT illustrated by the significant correlation between epicardial fat thickness and VAT cross-sectional areas at the level of L4 [16] in initial echocardiographic studies. This suggests that in predisposed subjects, growth of both fat depots may be the consequence of the activation of similar molecular pathways [17]. This assumption is reinforced by the demonstration that CAT and VAT share some properties such as low-grade chronic inflammation and production of pro-and anti-inflammatory cytokines [18, 19]. Increased epicardial adipose tissue thickness in echocardiography has been related to incident cardiovascular disease and mortality in patients with type-2 diabetes over 4.7 years of follow-up independent of traditional risk factors [20].

CT is a prime imaging modality in the present setting by offering a high spatial resolution coverage of the chest with precise exploration of the lungs but also excellent native fat to myocardium contrast not requiring contrast injection and has largely been used to quantify pericardiac fat thickness, area, and volume. An advantage of CT over echocardiography is the volumetric measurement of epi- and pericardial hence total cardiac adipose tissue irrespective of body morphology and size which hampers, together with high operator dependence, ultrasound-based assessment. The use of an automated 3D approach, although it cannot yet reliably separate pericardial from epicardial fat, has the advantage of high reproducibility. Increased CAT has been found using CT in diabetic patients compared to non-diabetic patients [21]. Furthermore, CT has become a landmark technique for non-invasive coronary artery atherosclerotic plaque imaging with increasing data showing a relation between CAT volume measured in CT, but not mediastinal fat, with both calcified and non-calcified atherosclerosis and adverse cardiovascular events [22, 23]. Pericoronary fat assessed by CT and analysis of fat attenuation index related to inflammation has also been shown to correlate to cardiovascular events strongly emphasizing the potential proatherogenic and proinflammatory role of CAT and complex interplay with the arterial wall and underlying myocardium [24, 25]. Cardiovascular involvement and adverse outcome has been largely reported in COVID-19 including acute coronary syndrome, myocarditis, arrhythmia and heart failure with hypertension, diabetes and obesity being the most prevalent risk factors (> 20%) [26].

**Fig. 4:**
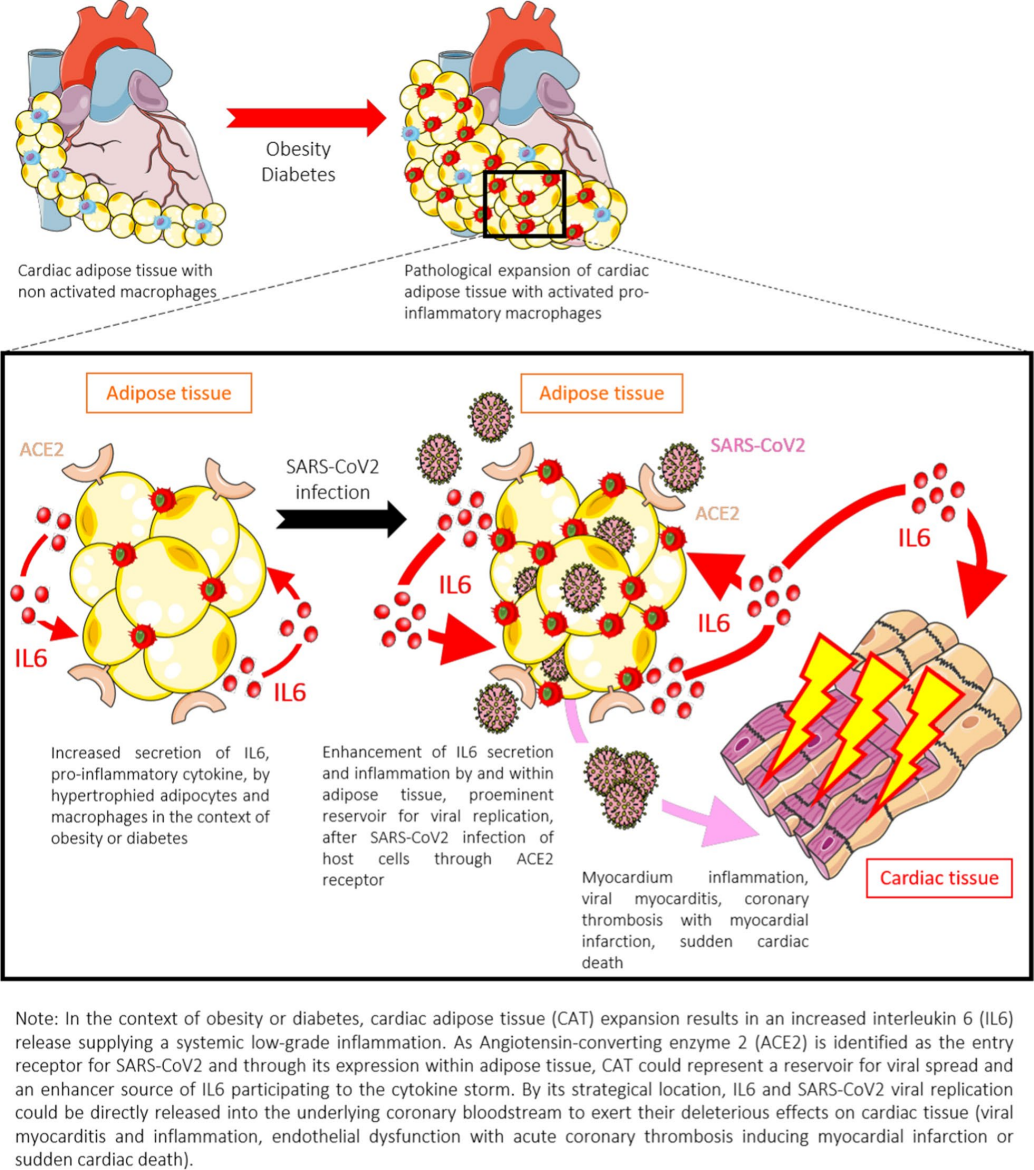
Schematic illustration of potential pathophysiological mechanisms relating cardiac adiposity to adverse outcome in diabetic COVID-19 patients

Subsequently, we sought to address CAT contribution in the severity of COVID-19 owing to the susceptibility of this tissue for SARS-CoV2 entry and replication. Potential pathological pathways linking CAT to COVID-19-related cardiovascular injury is summarized in Fig. 4. SARS-CoV-2 binding to angiotensin-converting enzyme 2 (ACE2) receptor is a critical step to mediate virus entry into target cells. ACE2 gene expression is higher in VAT and CAT than in lung tissue, reinforcing the important role of both fat depots in COVID-19 pathophysiology [27]. Additionally, SARS-CoV2 receptor richness in CAT may play an important role in the pathophysiology of CAT-mediated myocardial dysfunction and death in the diabetic and obese population and has been considered as a new marker of severe outcome during ischemic events [28]. Thus, beyond this deleterious role, CAT may be a prominent viral reservoir and an enhancer of pro-inflammatory cytokines secretion, notably IL-6, at a systemic level participating to the SARS-CoV-2-induced cytokine storm. Thus, as discussed recently [29] these data strongly suggested that, beyond BMI, ectopic fat such as VAT and CAT may have considerable importance in determining risk of adverse outcome in COVID-19.

We developed and propose a simple risk score, which was able to predict short-term mortality or ICU requirement in COVID-19 patients with type-II diabetes, a condition at high risk of deleterious and lethal evolution. In comparison to other scores (see Additional file 1: Table S1), our predictive score had at least equivalent or better predictive performance for short term mortality and/or ICU requirement and can be determined quickly after hospital admission, using only two parameters potentially routinely available: IL-6 and CATi alleviating the need for many other expensive biological measurements (see Additional file 1: Table S1). As an example, Bennouar et al. validate a risk score including age and 6 biological variables (blood urea nitrogen, LDH, NLR, CRP, albumin and natremia) combined into an ordinal risk score consisting of seven consecutive levels (S0-S7), survival duration decreasing gradually with increasing risk score levels [30]. Another score has been developed by Liang et al. to predict admission to ICU, invasive ventilation, and death including 10 variables (chest radiographic abnormality, age, hemoptysis, dyspnea, level of consciousness, number of comorbidities, cancer history, neutrophil-to-lymphocyte ratio, lactate dehydrogenase and direct bilirubin) [31]. But, this model was challenged since the process of variable selection was not clearly reported [32]. Additionally, Boran Hao et al. created three separate models that can predict hospitalization, ICU admission, and the need for mechanical ventilation, based on patient characteristics, clinical symptoms, laboratory results (10 variables) and chest X-ray [33] leading to complex score calculation requiring an online application. Consequently, some limitations for a routine use by clinicians in a context of emergency can be anticipated using this score and others [18, 31, 34].

Our results suggest that CATi is an individual predictor of risk of COVID-19 severity and early death as it represents the cardiovascular reservoir of both virus entry and pro-inflammatory cytokine release whereas IL-6 is an instantaneous measure of the systemic inflammation level. In that regard, while related, both measures are complementary. In our cohort, sensitivity and specificity analyses indeed suggest that the combination of CATi and IL-6 may be a more sensitive and specific predictor of severe outcome in patients with type-II diabetes than when these imaging and inflammation parameters are considered individually. Importantly, AUCROC for predicting outcomes were similar to other previously published scores. Consequently, CATi and IL-6 determination at admission could help physicians to better identify diabetic patients with a potentially severe and lethal course, irrespective of obesity. Early consideration for corticosteroid therapy and possibly anti-IL-6 drugs may improve the adverse COVID-19 prognosis in diabetic patients. Our results suggest that diabetic patients with high CAT volume at admission, *a fortiori* associated with high IL-6 levels could be a relevant target population to promptly initiate preventive anti-inflammatory therapies.

The results of the present study should be tempered by its limitations. First, the sample size was limited because we included only patients admitted to a single highly specialized center, and these results must be externally validated before they can be generalized to other hospitals. It is evident that factors other than those associated with the need for mechanical ventilation cannot be evaluated using the inflammatory markers in our scoring system. In particular, the administration of therapies, such as glucocorticoids and anti-cytokine therapies during hospitalization, could modify mortality, but this was outside the scope of our study. Broader, more in-depth studies are urgently required to develop more efficient risk rating systems than the current ones.

## Conclusions

Increased cardiac adipose tissue index and IL-6 relate significantly to early mortality and ICU requirement in COVID-19 patients with diabetes. CATi and IL-6 measurement at admission and the use of a composite score could help physicians to better identify such patients with a potentially severe and lethal course, irrespective of obesity and consider early preventive anti-inflammatory therapies.

## Supplementary Information

**Additional file 1: Table S1.** Summary of main studies to determine a prognostic score in COVID-19 patients.

## Data Availability

The datasets generated during and/or analysed during the current study are not publicly available due to storage under the umbrella of the Paris Academic Hospitals Group Assistance Publique-Hôpitaux de Paris EDS research data storage warehouse but may be available from the corresponding institution on reasonable request.

## Abbreviations

COVID-19: Coronavirus disease 19
AUC: Area under the curve
BMI: Body mass index
CAT: Cardiac adipose tissue
CT: Computed tomography
EAT: Epicardial adipose tissue
ICU: Intensive care unit
CMU: Conventional medical care unit
IL-6: Interleukin-6
IQR: Interquartile range
OR: Odds ratio
SARS-CoV2: Severe acute respiratory syndrome coronavirus 2
VAT: Visceral adipose tissue

## References

[1] Popkin BM, Du S, Green WD, Beck MA, Algaith T, Herbst CH (2020). Individuals with obesity and COVID-19: a global perspective on the epidemiology and biological relationships. Obes Rev.

[2] Eslami V, Abrishami A, Zarei E, Khalili N, Baharvand Z, Sanei-Taheri M (2020). The association of CT-measured cardiac indices with lung involvement and clinical outcome in patients with COVID-19. Acad Radiol.

[3] Chandarana H, Dane B, Mikheev A, Taffel MT, Feng Y, Rusinek H (2020). Visceral adipose tissue in patients with COVID-19: risk stratification for severity. Abdom Radiol..

[4] Iacobellis G (2009). Relation of epicardial fat thickness to right ventricular cavity size in obese subjects. Am J Cardiol.

[5] Katz JN, Sinha SS, Alviar CL, Dudzinski DM, Gage A, Brusca SB (2020). COVID-19 and disruptive modifications to cardiac critical care delivery: JACC review topic of the week. J Am Coll Cardiol.

[6] Petrilli CM, Jones SA, Yang J, Rajagopalan H, O’Donnell L, Chernyak Y (2020). Factors associated with hospital admission and critical illness among 5279 people with coronavirus disease 2019 in New York City: prospective cohort study. BMJ.

[7] Giacomelli A, Ridolfo AL, Milazzo L, Oreni L, Bernacchia D, Siano M (2020). 30-day mortality in patients hospitalized with COVID-19 during the first wave of the Italian epidemic: a prospective cohort study. Pharmacol Res.

[8] Sattar N, Gaw A, Scherbakova O, Ford I, O’Reilly DS, Haffner SM (2003). Metabolic syndrome with and without C-reactive protein as a predictor of coronary heart disease and diabetes in the West of Scotland Coronary Prevention Study. Circulation.

[9] Pranata R, Lim MA, Huang I, Yonas E, Henrina J, Vania R (2021). Visceral adiposity, subcutaneous adiposity, and severe coronavirus disease-2019 (COVID-19): Systematic review and meta-analysis. Clin Nutr ESPEN.

[10] Schipper HS, Prakken B, Kalkhoven E, Boes M (2012). Adipose tissue-resident immune cells: key players in immunometabolism. Trends Endocrinol Metab.

[11] Neeland IJ, Ross R, Després J-P, Matsuzawa Y, Yamashita S, Shai I (2019). Visceral and ectopic fat, atherosclerosis, and cardiometabolic disease: a position statement. Lancet Diabetes Endocrinol.

[12] Lazzeri C, Bonizzoli M, Batacchi S, Di Valvasone S, Chiostri M, Peris A (2021). The prognostic role of hyperglycemia and glucose variability in covid-related acute respiratory distress Syndrome. Diabetes Res Clin Pract.

[13] Holman N, Knighton P, Kar P, O’Keefe J, Curley M, Weaver A (2020). Risk factors for COVID-19-related mortality in people with type 1 and type 2 diabetes in England: a population-based cohort study. Lancet Diabetes Endocrinol.

[14] Cariou B, Hadjadj S, Wargny M, Pichelin M, Al-Salameh A, Allix I (2020). Phenotypic characteristics and prognosis of inpatients with COVID-19 and diabetes: the CORONADO study. Diabetologia.

[15] Bouazizi K, Zarai M, Dietenbeck T, Aron-Wisnewsky J, Clément K, Redheuil A (2021). Abdominal adipose tissue components quantification in MRI as a relevant biomarker of metabolic profile. Magn Reson Imaging.

[16] Bertaso AG, Bertol D, Duncan BB, Foppa M (2013). Epicardial fat: definition, measurements and systematic review of main outcomes. Arq Bras Cardiol.

[17] Iacobellis G, Assael F, Ribaudo MC, Zappaterreno A, Alessi G, Di Mario U (2003). Epicardial fat from echocardiography: a new method for visceral adipose tissue prediction. Obes Res.

[18] Baker KF, Hanrath AT, van der SchimLoeff I, Kay LJ, Back J, Duncan CJ (2021). National Early Warning Score 2 (NEWS2) to identify inpatient COVID-19 deterioration: a retrospective analysis. Clin Med.

[19] Cheng K-H, Chu C-S, Lee K-T, Lin T-H, Hsieh C-C, Chiu C-C (2008). Adipocytokines and proinflammatory mediators from abdominal and epicardial adipose tissue in patients with coronary artery disease. Int J Obes (Lond).

[20] Christensen RH, von Scholten BJ, Hansen CS, Jensen MT, Vilsbøll T, Rossing P (2019). Epicardial adipose tissue predicts incident cardiovascular disease and mortality in patients with type 2 diabetes. Cardiovasc Diabetol.

[21] Milanese G, Silva M, Bruno L, Goldoni M, Benedetti G, Rossi E (2019). Quantification of epicardial fat with cardiac CT angiography and association with cardiovascular risk factors in symptomatic patients: from the ALTER-BIO (Alternative Cardiovascular Bio-Imaging markers) registry. Diagn Interv Radiol.

[22] Konishi M, Sugiyama S, Sato Y, Oshima S, Sugamura K, Nozaki T (2010). Pericardial fat inflammation correlates with coronary artery disease. Atherosclerosis.

[23] Mahabadi AA, Balcer B, Dykun I, Forsting M, Schlosser T, Heusch G (2017). Cardiac computed tomography-derived epicardial fat volume and attenuation independently distinguish patients with and without myocardial infarction. PLoS ONE.

[24] Antonopoulos AS, Sanna F, Sabharwal N, Thomas S, Oikonomou EK, Herdman L (2017). Detecting human coronary inflammation by imaging perivascular fat. Sci Transl Med..

[25] Oikonomou EK, Marwan M, Desai MY, Mancio J, Alashi A, Centeno EH (2018). Non-invasive detection of coronary inflammation using computed tomography and prediction of residual cardiovascular risk (the CRISP CT study): a post-hoc analysis of prospective outcome data. Lancet.

[26] Pellicori P, Doolub G, Wong CM, Lee KS, Mangion K, Ahmad M (2021). COVID-19 and its cardiovascular effects: a systematic review of prevalence studies. Cochrane Database Syst Rev.

[27] Al-Benna S (2020). Association of high level gene expression of ACE2 in adipose tissue with mortality of COVID-19 infection in obese patients. Obes Med..

[28] Flinn B, Royce N, Gress T, Chowdhury N, Santanam N (2021). Dual role for angiotensin-converting enzyme 2 in Severe Acute Respiratory Syndrome Coronavirus 2 infection and cardiac fat. Obes Rev.

[29] Zhao Y, Chen M, Yang X, Zhao L. COVID-19 letter to the editor: Epicardial fat inflammation as possible enhancer in COVID-19? Metabolism Clinical and Experimental [Internet]. 2021. https://www.metabolismjournal.com/article/S0026-0495(21)00022-6/abstract. Accessed 8 Feb 8. 10.1016/j.metabol.2021.154722 PMC7857989 33548252

[30] Bennouar S, BachirCherif A, Kessira A, Bennouar D-E, Abdi S (2021). Development and validation of a laboratory risk score for the early prediction of COVID-19 severity and in-hospital mortality. Intensive Crit Care Nurs.

[31] Liang W, Liang H, Ou L, Chen B, Chen A, Li C (2020). Development and validation of a clinical risk score to predict the occurrence of critical illness in hospitalized patients with COVID-19. JAMA Intern Med.

[32] Gu H-Q, Wang J (2021). Prediction models for COVID-19 need further improvements. JAMA Intern Med.

[33] Hao B, Sotudian S, Wang T, Xu T, Hu Y, Gaitanidis A (2020). Early prediction of level-of-care requirements in patients with COVID-19. Elife.

[34] Kim DH, Park HC, Cho A, Kim J, Yun K-S, Kim J (2021). Age-adjusted Charlson comorbidity index score is the best predictor for severe clinical outcome in the hospitalized patients with COVID-19 infection. Medicine.

