# Supplementary material for: Cardiac adipose tissue volume and IL-6 level at admission are complementary predictors of severity and short-term mortality in COVID-19 diabetic patients
Source: Cardiovasc Diabetol. 2021 Aug 12;20:165. doi: 10.1186/s12933-021-01327-1 (PMC8358546; doi:10.1186/s12933-021-01327-1)
Supplement: Supplementary file 1 — Additional file 1: Table S1. Summary of main studies to determine a prognostic score in COVID-19 patients. [file 12933_2021_1327_MOESM1_ESM.docx]

**Addiitonal file 1: Table S1: Summary of main studies to determine a prognostic score in COVID-19 patients**

| **Main authors** | **Study type** | **Study population** | **Studied outcomes and explanation variables** | **Main results** |
| --- | --- | --- | --- | --- |
| Venturi S et al, Rev Esp Anestesiol Reanim. 2020 Oct;67(8):425-4372021 | retrospective | 22 consecutive patients: 8 died | Lymphocyte  platelet  Prothrombin time |  |
| Ferrando C, et al. Rev Esp Anestesiol Reanim. 2020 Oct;67(8):425-437. | Prospective, multicentre | 663 patients, ICU mortality: 31% 203 patients | Death:  Age  APACHE II score  acute kidney injury  cardiac arrest  septic shock |  |
| Martin S et al, Scand J Clin Lab Invest. 2021 May 11:1-8. | retrospective cohort | 264 patients  critical illness: 62 (23.5%)  invasive ventilation 21 (8.0%)  ICU admission 34 (12.9%) overall mortality 39 cases (14.8%) | age  CRP  lymphocytes count  CK  creatinine | AUC : 0.850  sensitivity of 65% specificity of 87% |
| Amezcua-Guerra LM et al, Inflamm Res. 2021 | prospective | 100 patients | Risk of mechanical ventilation:  inflammation (WBC count≥9.3 × 103 cells/μL,  CRP level ≥13.0 mg/L, serum albumin level≤ 3.6 g/dL)  D-dimer > 574 ng/mL  troponin I ≥ 6.7 ng/mL |  |
| UCAN ES et al, Respir Med Res 2021 | Retrospective | 298 patients | **A-DROP Score**  **A**ge (male ≥ 70 years, female ≥ 75 years); **D**ehydration (blood urea nitrogen (BUN) ≥ 210 mg/L);  **R**espiratory failure (SaO_2_ ≤ 90% or PaO_2_ ≤ 60 mm Hg);  **O**rientation disturbance (confusion);  (v) low blood **P**ressure (systolic blood pressure ≤ 90 mm Hg)  CURB-65 score  Confusion  Urea >7 mmol/l  Respiratory rate ⩾30/min, low systolic(<90 mm Hg) or diastolic (⩽60 mm Hg) Blood pressure)  age ⩾65 years | Mortality AUC of A-DROP score : 0,875  HR: 2.31, 95%CI: 1.62–3.30  Mortality AUC of CURB-65 score : 0,737  HR: 2.01, 95% CI: 1.57–2.96 |
| Baker KF et al, Clin Med (Lond) 2021 Mar | restrospective | 294 | **Prediction** initiation of respiratory support, admission to intensive care, initiation of end of life care, or in-hospital death.  **NEWS2 score**  respiratory rate,  hypercapnic respiratory failure,  supplemental oxygen,  body temperature,  systolic blood pressure,  pulse rate  level of consciousness. | sensitivity 0.98 (95% CI 0.96-1.00),  specificity 0.28 (0.21-0.35), positive predictive value (PPV) 0.53 (0.47-0.59),  negative predictive value (NPV) 0.96 (0.90-1.00). |
| Liang W et al, JAMA Intern Med 2020 Aug | retrospective | 1590 | **Prediction of** admission to the intensive care unit, invasive ventilation, or death.  **COVID-GRAM risk score =**  (X-ray abnormality × 27.1464) + (age × 0.6139) +  (hemoptysis × 33.6210) + (dyspnoea × 14.0569) +  (unconsciousness × 34.4617) + (number of comorbidities × 10.3826) + (cancer history × 31.2211) + (neutrophil/lymphocyte ratio, N/L × 1.25) + (lactate dehydrogenase, LDH × 0.0534) + (direct bilirubin × 3.0605). | AUC 0.88 (95% CI, 0.85-0.91) |
| Vittorio De Socio G et al, Infection 2021 May | Retrospective, multicentric | 121 | Comparison of COVID-GRAM score and NEWS2 scores for admission to the intensive care unit,  invasive ventilation,  or death. | NEWS2 AUROC curve 0.87 (standard error, SE 0.03; 95% CI 0.80–0.93; p < 0.0001).  COVID-GRAM score AUROC curve 0.77 (SE 0.04; 95% CI 0.68–0.85; p < 0.0001)  NEWS2 better predicted severe COVID-19 |
| Bellos I et al, Int J Clin Pract  . 2021 Apr | Prospective | 67 | Prediction of intensive care unit admission  gender, presence of hypertension and diabetes mellitus, fever, shortness of breath, serum glucose, aspartate aminotransferase, lactate dehydrogenase, C-reactive protein and fibrinogen | AUC 97.1%  sensitivity 92.3%  specificity 93.3% |
| Stachel A, et al. BMJ Health Care Inform. 2021 May | Retrospective | 3395 | Prediction of death  Oximetry, respirations, blood urea nitrogen, lymphocyte per cent, calcium, troponin and neutrophil percentage | AUCROC 0.83 |
| Kim DH et al. Medicine (Baltimore). 2021 May | Retrospective | 5621 | Prediction of death  dyspnea (hazard ratio [HR] 2.88, 95% confidence interval [CI] 2.16-3.83), low body mass index < 18.5 kg/m2 (HR 2.36, CI 1.49-3.75), lymphopenia (<0.8 x109/L) (HR 2.15, CI 1.59-2.91), thrombocytopenia (<150.0 x109/L) (HR 1.29, CI 0.94-1.78), anemia (<12.0 g/dL) (HR 1.80, CI 1.33-2.43), and male sex (HR 1.76, CI 1.32-2.34) | AUC 0.93 |
| Duca A et al. Emerg Med Pract 2020 |  |  | **BCRSS (**Brescia-COVID Respiratory Severity Scale ) for  prediction of intensive care unit (ICU) admissions and death:  wheezing or unable to speak in full sentences while at rest/with minimal effort (replaced with patient reporting shortness of breath, given the retrospective nature of this study), respiratory rate >22, oxygen saturation (SpO_2_) <90%, and repeat chest X-ray with significant worsening (defined as bilateral or diffuse infiltrates) |  |
| Haimovich A.D.et al. Ann Emerg Med. 2020; | Retrospective | 1792 | Quick COVID-19 Severity Index for  prediction of respiratory failure within 24 hours of admission as defined by oxygen requirement of greater than 10 L/min by low-flow device, high-flow device, noninvasive or invasive ventilation, or death  nasal cannula flow rate, respiratory rate, and minimum documented pulse oximetry | AUCROC 0.81 [95% confidence interval {CI} 0.73 to 0.89]) |
| Gude-Sampedro F et al. Int J Epidemiol. 2021 Mar | Retrospective | 2292 | Prediction score for admission to intensive care unit (ICU) and mortality  age, gender and chronic comorbidities such as cardiovascular disease, diabetes, obesity, hypertension, chronic obstructive pulmonary disease, asthma, liver disease, chronic kidney disease and haematological cancer. | AUCROC admission to ICU [AUC 0.83 (95%CI 0.81, 0.85)]  AUCROC death [AUC 0.89 (95%CI 0.88, 0.90)] |
| Zhao Z et al. PLoS One. 2020 | Retrospective | 641 | Prediction of ntensive care unit (ICU) admission  lactate dehydrogenase, procalcitonin, pulse oxygen saturation, smoking history, and lymphocyte count  Prediction of mortality  heart failure, procalcitonin, lactate dehydrogenase, chronic obstructive pulmonary disease, pulse oxygen saturation, heart rate, and age | AUC of 0.74 ([95% CI, 0.63-0.85], p = 0.001) for predicting ICU admission and 0.83 ([95% CI, 0.73-0.92], p<0.001) |
| Allenbach Y et al. PLoS One. 2020 | Prospective | 152 | Prediction of intensive care unit (ICU) transfer or death at day 14 (D14), of being discharge alive and severe status at D14 (remaining with ventilation, or death)  Older age (OR 2.61, 95% CI 0.96-7.10), poorer respiratory presentation (OR 4.04 per 1-point increment on World Health Organization (WHO) clinical scale, 95% CI 1.76-9.25), higher CRP-level (OR 1.63 per 100mg/L increment, 95% CI 0.98-2.71) and lower lymphocytes count (OR 0.36 per 1000/mm3 increment, 95% CI 0.13-0.99) were associated with an increased risk of ICU requirement or death | sensitivity 97% (95% CI 94–100), specificity 94% (95% CI 89–98) |

1. Venturini S, Orso D, Cugini F, Crapis M, Fossati S, Callegari A, Pellis T, Tomasello DC, Tonizzo M, Grembiale A, D'Andrea N, Vetrugno L, Bove T. Artificial neural network model from a case series of COVID-19 patients: a prognostic analysis. Acta Biomed. 2021 May 12;92(2):e2021202.
2. Ferrando C, Mellado-Artigas R, Gea A, Arruti E, Aldecoa C, Bordell A, Adalia R, Zattera L, Ramasco F, Monedero P, Maseda E, Martínez A, Tamayo G, Mercadal J, Muñoz G, Jacas A, Ángeles G, Castro P, Hernández-Tejero M, Fernandez J, Gómez-Rojo M, Candela Á, Ripollés J, Nieto A, Bassas E, Deiros C, Margarit A, Redondo FJ, Martín A, García N, Casas P, Morcillo C, Hernández-Sanz ML; de la Red de UCI Española para COVID-19. Patient characteristics, clinical course and factors associated to ICU mortality in critically ill patients infected with SARS-CoV-2 in Spain: A prospective, cohort, multicentre study. Rev Esp Anestesiol Reanim. 2020 Oct;67(8):425-437.
3. Martin S, Fuentes S, Sanchez C, Jimenez M, Navarro C, Perez H, Salamanca E, Santotoribio JD, Bobillo J, Giron JA, Gonzalez J, Garrido JM, Liro J, Guerrero JM, Sanchez-Pozo MC, Sanchez-Margalet V, Leon-Justel A. Development and validation of a laboratory-based risk score to predict the occurrence of critical illness in hospitalized patients with COVID-19. Scand J Clin Lab Invest. 2021 May 11:1-8. doi: 10.1080/00365513.2020.1847313.
4. Amezcua-Guerra LM, Audelo K, Guzmán J, Santiago D, González-Flores J, García-Ávila C, Torres Z, Baranda-Tovar F, Tavera-Alonso C, Sandoval J, González-Pacheco H. A simple and readily available inflammation-based risk scoring system on admission predicts the need for mechanical ventilation in patients with COVID-19. Inflamm Res. 2021 May 10:1–12. doi: 10.1007/s00011-021-01466-x.
5. Ucan ES, Ozgen Alpaydin A, Ozuygur SS, Ercan S, Unal B, Sayiner AA, Ergan B, Gokmen N, Savran Y, Kilinc O, Avkan Oguz V; DEU COVID Study Group. Pneumonia severity indices predict prognosis in coronavirus disease-2019. Respir Med Res. 2021 Apr 27;79:100826.
6. Baker KF, Hanrath AT, van der Loeff SI, Kay LJ, Back J, Duncan CJ. National early warning score 2 (NEWS2) to identify inpatient COVID-19 deterioration: a retrospective analysis. *Clin Med.*2021;21:84. doi: 10.7861/clinmed.2020-0688.
7. Liang W, Liang H, Ou L, Chen B, Chen A, Li C, Li Y, Guan W, Sang L, Lu J, Xu Y, Chen G, Guo H, Guo J, Chen Z, Zhao Y, Li S, Zhang N, Zhong N, He J; China Medical Treatment Expert Group for COVID-19. Development and Validation of a Clinical Risk Score to Predict the Occurrence of Critical Illness in Hospitalized Patients With COVID-19. JAMA Intern Med. 2020 Aug 1;180(8):1081-1089.
8. De Socio GV, Gidari A, Sicari F, Palumbo M, Francisci D. National Early Warning Score 2 (NEWS2) better predicts critical Coronavirus Disease 2019 (COVID-19) illness than COVID-GRAM, a multi-centre study. Infection. 2021 May 10:1–6.
9. Bellos I, Lourida P, Argyraki A, Korompoki E, Zirou C, Kokkinaki I, Pefanis A. Development of a novel risk score for the prediction of critical illness amongst COVID-19 patients. Int J Clin Pract. 2021 Apr;75(4):e13915.
10. Stachel A, Daniel K, Ding D, Francois F, Phillips M, Lighter J. Development and validation of a machine learning model to predict mortality risk in patients with COVID-19. BMJ Health Care Inform. 2021 May;28(1):e100235.
11. Kim DH, Park HC, Cho A, Kim J, Yun KS, Kim J, Lee YK. Age-adjusted Charlson comorbidity index score is the best predictor for severe clinical outcome in the hospitalized patients with COVID-19 infection. Medicine (Baltimore). 2021 May 7;
12. Duca A, Piva S, Focà E, Latronico N, Rizzi M. Calculated Decisions: Brescia-COVID Respiratory Severity Scale (BCRSS)/Algorithm. Emerg Med Pract 2020;22(5 Suppl):CD1-CD2.
13. Haimovich A.D., Ravindra N.G., Stoytchev S., Young H.P., Wilson F.P., van Dijk D. Development and validation of the quick covid-19 severity index: a prognostic tool for early clinical decompensation. *Ann Emerg Med.*2020;76(4):442–453.
14. Gude-Sampedro F, Fernández-Merino C, Ferreiro L, Lado-Baleato Ó, Espasandín-Domínguez J, Hervada X, Cadarso CM, Valdés L. Development and validation of a prognostic model based on comorbidities to predict COVID-19 severity: a population-based study. Int J Epidemiol. 2021 Mar 3;50(1):64-74.
15. Zhao Z, Chen A, Hou W, Graham JM, Li H, Richman PS, Thode HC, Singer AJ, Duong TQ. Prediction model and risk scores of ICU admission and mortality in COVID-19. PLoS One. 2020 Jul 30;15(7):e0236618.
16. Allenbach Y, Saadoun D, Maalouf G, Vieira M, Hellio A, Boddaert J, Gros H, Salem JE, Resche Rigon M, Menyssa C, Biard L, Benveniste O, Cacoub P; DIMICOVID. Development of a multivariate prediction model of intensive care unit transfer or death: A French prospective cohort study of hospitalized COVID-19 patients. PLoS One. 2020 Oct 19;15(10):e0240711.
